# Vitiligo: A Possible Model of Degenerative Diseases

**DOI:** 10.1371/journal.pone.0059782

**Published:** 2013-03-26

**Authors:** Barbara Bellei, Angela Pitisci, Monica Ottaviani, Matteo Ludovici, Carlo Cota, Fabiola Luzi, Maria Lucia Dell'Anna, Mauro Picardo

**Affiliations:** 1 Laboratory of Cutaneous Physiopathology, San Gallicano Dermatologic Institute, Istituto Di Ricovero e Cura a Carattere Scientifico, Rome, Italy; 2 Dermatopathology Unit, San Gallicano Dermatological Institute, Istituto Di Ricovero e Cura a Carattere Scientifico, Rome, Italy; 3 Struttura Complessa di Medicina Preventiva delle Migrazioni, del Turismo e di Dermatologia Tropicale, San Gallicano Dermatological Institute, Istituto Di Ricovero e Cura a Carattere Scientifico, Rome, Italy; German Cancer Research Center, Germany

## Abstract

Vitiligo is characterized by the progressive disappearance of pigment cells from skin and hair follicle. Several *in vitro* and *in vivo* studies show evidence of an altered redox status, suggesting that loss of cellular redox equilibrium might be the pathogenic mechanism in vitiligo. However, despite the numerous data supporting a pathogenic role of oxidative stress, there is still no consensus explanation underlying the oxidative stress-driven disappear of melanocytes from the epidermis. In this study, *in vitro* characterization of melanocytes cultures from non-lesional vitiligo skin revealed at the cellular level aberrant function of signal transduction pathways common with neurodegenerative diseases including modification of lipid metabolism, hyperactivation of mitogen-activated protein kinase (MAPK) and cAMP response element-binding protein (CREB), constitutive p53-dependent stress signal transduction cascades, and enhanced sensibility to pro-apoptotic stimuli. Notably, these long-term effects of subcytotoxic oxidative stress are also biomarkers of pre-senescent cellular phenotype. Consistent with this, vitiligo cells showed a significant increase in p16 that did not correlate with the chronological age of the donor. Moreover, vitiligo melanocytes produced many biologically active proteins among the senescence-associated secretory phenotype (SAPS), such as interleukin-6 (IL-6), matrix metallo proteinase-3 (MMP3), cyclooxygenase-2 (Cox-2), insulin-like growth factor-binding protein-3 and 7 (IGFBP3, IGFBP7). Together, these data argue for a complicated pathophysiologic puzzle underlying melanocytes degeneration resembling, from the biological point of view, neurodegenerative diseases. Our results suggest new possible targets for intervention that in combination with current therapies could correct melanocytes intrinsic defects.

## Introduction

Vitiligo is an acquired cutaneous skin and less frequently hair disease characterized by declining melanocyte function and depigmentation with an estimated prevalence of 0.5–1% in most populations [1]. Depigmentation results in irregular white patches, initially small, that often gradually enlarge and change shape and this process is a chronic with a highly variable course. In contrast to other prevalent skin diseases vitiligo has a near orphan status for drug development. Several mechanisms of melanocyte degeneration have been presented, including autoimmunity [2], autocytotoxic/metabolic mechanism [3]–[4], and impaired melanocyte migration and/or proliferation [5]. Studies have also pointed to a significant role of genetic susceptibility to vitiligo [6]. Since none of these processes alone are sufficient to fully explain the mechanisms of the disease, and all of the proposed mechanisms are not mutually exclusive, the convergence theories have been formulated combining biochemical, environmental and immunological events, in a permissive genetic milieu [7]. Despite considerable efforts, there are few data in the literature on *in vitro* studies of vitiligo epidermal melanocytes, mainly due to the fact that cells from normally pigmented skin of vitiligo patients demonstrate reduced initial seeding and proliferation capacity compared to healthy adult human skin [8]–[9].

Several investigators have proved the presence of oxidative stress in cultured melanocytes coupled with an increased susceptibility to pro-oxidant agents [3], [10]–[11]. *In vivo* oxidative stress has been attributed to a massive accumulation of H_2_O_2_ in vitiligo skin, which is associated with impaired catalase and glutathione peroxidase activities [12]. Moreover, there are several lines of evidence for systemic oxidative stress, including alteration of peripheral blood antioxidant patterns and oxidative DNA damage [13]–[14]. Impairment of mitochondrial complex I respiratory chain and increased expression of malate dehydrogenase suggest that in vitiligo mitochondria could be the site of uncontrolled ROS production [13].

Recently, nuclear factor E2-related factor 2 (Nrf2), one of the most critical antioxidant enzymatic systems, and its downstream target genes were found increased in vitiligo non-lesional skin biopsies, suggesting that a consistently higher Nrf2-dependent transcriptional activity is required for the maintenance of redox homeostasis in disease-free epidermis [15]. Consistent with the idea that persistent intracellular oxidative stress burdens vitiligo skin, overexpression of p53 protein has been reported in both lesional and non-lesional epidermis of patients with vitiligo [16]–[17]. However, despite numerous data supporting a pathogenic role of oxidative stress, there is still no consensus explanation underlying the oxidative stress-driven disappearance of melanocytes from the epidermis [18]. Even though increased susceptibility to the toxic effects of chemical oxidants or UVB has been reported in several *in vitro* studies [3], [9], [19] there are no convincing data demonstrating the occurrence of melanocytes death by cytotoxicity or apoptosis in vitiligo skin *in vivo*. Biopsy material from established lesions contains no or few melanocytes, and it is difficult to capture the essence of the melanocytes lost. However, it is also possible that persistent stress-induced cell damage might interfere with vitiligo melanocytes vitality and functionality by initiating a senescence-driven melanocytes detachment.

Since many oxidative stress-related progressive or non-progressive chronic diseases are referred to as degenerative diseases we investigated the possibility that vitiligo melanocytes express common features with cells of neurodegenerative disorders. For most of these pathologies it has been demonstrated that sublethal oxidative stress and subsequent cellular alterations, including modification of lipid metabolism [20], impairment of the mitochondrial respiratory chain [21], dysfunction of intracellular signaling, and enhanced sensibility to pro-apoptotic stimuli [22], culminate in aging and cell degeneration [23]. Moreover, a premature senescent-like cell phenotype *in vivo* and *in vitro* has been linked to the degenerative diseases [24]. Some cells in aging organisms lose functionality: neurons, for example, lose the ability to form synapses despite cell bodies remaining viable [25]. A similar scenario is conceivable for vitiligo cells, because melanocytes are not necessarily completely absent in the depigmented lesions [26].

Here we report convincing evidence that vitiligo melanocytes have alterations of signal transduction pathways at the cellular and molecular level that altogether argue for a condition of stress-induced premature senescence-like phenotype supporting the classification of vitiligo among the degenerative diseases. Moreover, vitiligo non-lesional skin biopsies showed that results collected with cell culture experiments were not artificially-induced by culture conditions and confirmed the acquisition of p53-dependent pro-senescent phenotype.

## Materials And Methods

### Cell culture

VHM were isolated from skin biopsies obtained from a normally pigmented area in the gluteal or armpit regions. Patients (females = 6, male = 8) ranged from 7 to 56 years old (average 38.6 years). NHM were isolated from healthy individuals (females = 8, male = 8) who underwent plastic surgery and ranged from 15 to 75 years old (average 50.1 years). Cells were cultured in M254 medium supplemented with Human Melanocyte Growth Supplements (HMGS) (Cascade Biologics, Mansfield, UK) and used between passage 2 and 8. Institutional Research Ethics Committee (Istituti Fisioterapici Ospitalieri), approval was obtained to collect samples of human material for research. The Declaration of Helsinki Principles was followed and patients gave written informed consent. The study included one children participant and in this case his parents approved the written consent.

### Cell viability and proliferation

For cells proliferation assay cells were incubated with M254 containing HMGS or M254 alone for 24 h and then treated with *N*-acetyl-L-cystein (5 mM) (Sigma Aldrich, Milan, Italy), or pifithrin-α (5 µM) (Santa Cruz Biotechnology, Santa Cruz, CA, USA). Medium was replaced every 48 h and cells were left to growth according with experimental design before Trypan blue exclusion assay. For cell viability assay cells were exposed to *t*-BHP for 24 h and then incubated with 3-(4,5 dimethylthiazol)-2,5-diphenyl tetrazolium bromide (MTT) (Sigma Aldrich) for 2 h. After this time, the medium was removed and the resulting crystals were solubilized in DMSO. The absorbance was measured at 570 nm with a reference wavelength of 650 nm.

### Immunofluorescence

For indirect immunofluorescence experiments, cells were grown on coverslips and after treatment were fixed and permeabilized with methanol for 10 min at −20°C (catalase and SOD2 antibodies) or fixed in 3% paraformaldehyde in PBS for 15 min at room temperature and then permeabilized with 0.05% Trition X-100 in PBS for 5 min (p16, p53 and cyclinD1). Cells were rinsed in PBS and incubated for 2 h with mouse anti-catalase (Sigma, Milan, Italy), anti-p16 (Santa Cruz Biotechnology, USA) anti-p53 or anti-cyclinD1 (Dako, Milan, Italy) primary antibodies or rabbit anti-SOD2 (Stressgen Biothechnology (1∶300 in PBS). Cells were rinsed three times with PBS and incubated for 60 min with an Alexa-Fluor-488-conjugated goat anti-mouse IgG or goat anti-rabbit IgG (1∶800 in PBS) (Molecular Probe). Nuclei were stained with DAPI (Sigma, Milan Italy). For tissue immunofluorescence serial sections (3 µm) derived from formalin-fixed and paraffin-embedded blocks were dewaxed in xylene and rehydrated through graded ethanol to PBS. Tissue sections were incubated with the following primary antibodies: anti-p53 mouse monoclonal antibodies (1∶300) and anti-PML rabbit polyclonal antibodies (1∶400) plus anti-Tyrosinase antibody (1∶200) (Santa Cruz Bioechnology). Sections were then treated with conjugated with secondary antibodies AlexaFluor-488 chicken-anti-goat plus AlexaFluor-546 goat anti-rabbit or plus AlexaFluor-546 goat anti-mouse (1∶800) and stained with DAPI.

### Western blot analysis

Cell extracts were prepared with RIPA buffer containing proteases and phosphatases inhibitors. Proteins were separated on SDS-polyacrylamide gels, transferred to nitrocellulose membranes and then treated with p53, SOD2, catalase, PML and GADD45 (Santa Cruz Biotechnology, USA) antibodies. Anti-β-tubulin (Sigma Aldrich) was used to normalize protein content. Horseradish peroxide-conjugated goat anti-mouse or goat anti-rabbit secondary antibody complexes were detected by chemiluminescence (Santa Cruz Biotechnology).

### Determination of MMP3, IGFBP3 and IL-6 proteins

Quantitative measurement of the hIGFBP3, IL-6 (DRG Diagnostic, Marburg, Germany) and hMMP3 (Boster Biological Technology, Fremont, CA, USA) were used according with manufacture instructions. Due to a significant interference with HMGS components the level of IGFBP3 was determined using protein cell extracts whereas the level of MMP3 protein was measured using undiluted conditioned medium (96 h). Results were normalized for proteins concentration.

### Semi-quantitative RT-PCR

Total RNA was extracted using Aurum Total mini kit (BioRad, Milan Italy). cDNA was synthesized from 1 µg of total RNA using the FirstAid kit (Fermentas) and amplified in a reaction mixture containing iQSYBR Green Supermix (BioRad, Milan Italy) and 25 pmol of forward and reverse primers using an iQ5 Light Cycler (BioRad). All samples were run in triplicate, and relative expression was determined by normalizing results to β-actin mRNA. Due to the importance of the internal control gene chosen for sample normalization, comparative analyses were randomly carried out using two commonly used housekeeping genes: GAPDH and 18 S rRNA (data not shown). This confirmed that β-actin mRNA was a satisfactory reference gene. Sequences of primers can be found in Table S1.

### Flow cytometry analysis for MAPK phosphorylation

Melanocytes were fixed and permeabilized with Cytofix/Citoperm™and then stained with anti-p38-Alexa-Fluor-488-conjugated (pT180/pY182), anti-ERK-PE-conjugated (pT202/pY204) or anti-CREB-PE-conjugated (pSer133) antibodies (BD Bioscience, Erembodegem, Belgium) and then analyzed by flow cytometry using a FACSCalibur. Data from 1×10^4^ cells were acquired from each sample. Median Fluorescence Intensity (MFI) was evaluated on a linear scale.

### Assessment of intracellular reactive oxygen species

Production of ROS was assessed incubating cells with the fluorescent dye 2′7′-dichlorodihydrofluorescein diacetate (Sigma Aldrich, Milan) for 30 min at 37°C and 5% CO_2_ in the dark. Cells were washed with PBS and trypsinized, centrifuged at 1000 rpm, and then resuspended in PBS. Signals were measured by flow cytometry (BD Bioscience).

### Cholesterol membrane content measurement

Lipids were extracted twice in chloroform:methanol 2∶1 in the presence of butylated hydroxytoluene as an antioxidant and 5α-cholestane as an internal standard and centrifuged at 1800 rpm for 10 min. The lower phase was evaporated under a nitrogen stream. Oxysterol esters were hydrolyzed by means of 1M KOH in methanol. Combined organic extracts were evaporated under N_2_ flow and then directly silylated with N,O-bis-(trimethylsilyl)-trifluoroacetamide containing 1% trimethylchlorosilane as catalyst. An oven temperature gradient 180°C–250°C at 20 °C/min was used. Mass spectra were recorded in Electronic Impact and in SIM modality. The separation was performed by capillary column HP-5MS (30 m×0,250 nm×0,25 µm, Agilent Technologies INC) using helium as the carrier gas. Results are expressed as percentage ± SD considering NHMs content as 100.

### Immunohistochemistry

Serial sections (3 µm) derived from formalin-fixed and paraffin-embedded blocks were dewaxed in xylene and rehydrated through graded ethanol to PBS. Endogenous peroxidase activities were blocked by 0.03% hydrogen peroxide. Tissue sections were incubated with the following primary antibodies: anti-p53 mouse monoclonal antibodies (1∶300), anti-PML rabbit polyclonal antibodies (1∶400) and anti-GADD45 mouse monoclonal antibody (1∶300). Sections were then treated with peroxidase-labelled polymer conjugated with secondary antibodies (Dako), incubated with 3-amino-9-ethylcarbazole substrate chromogen (Dako) and counterstained with haematoxylin. Negative controls were obtained by omitting the primary antibodies.

### Statistical analysis

Student's *t*-test was used to assess statistical significance with thresholds of * p≤0.05 and ** p≤0.01.

## Results

### Intrinsic oxidative stress in vitiligo melanocytes cultures

**Figure 1:**
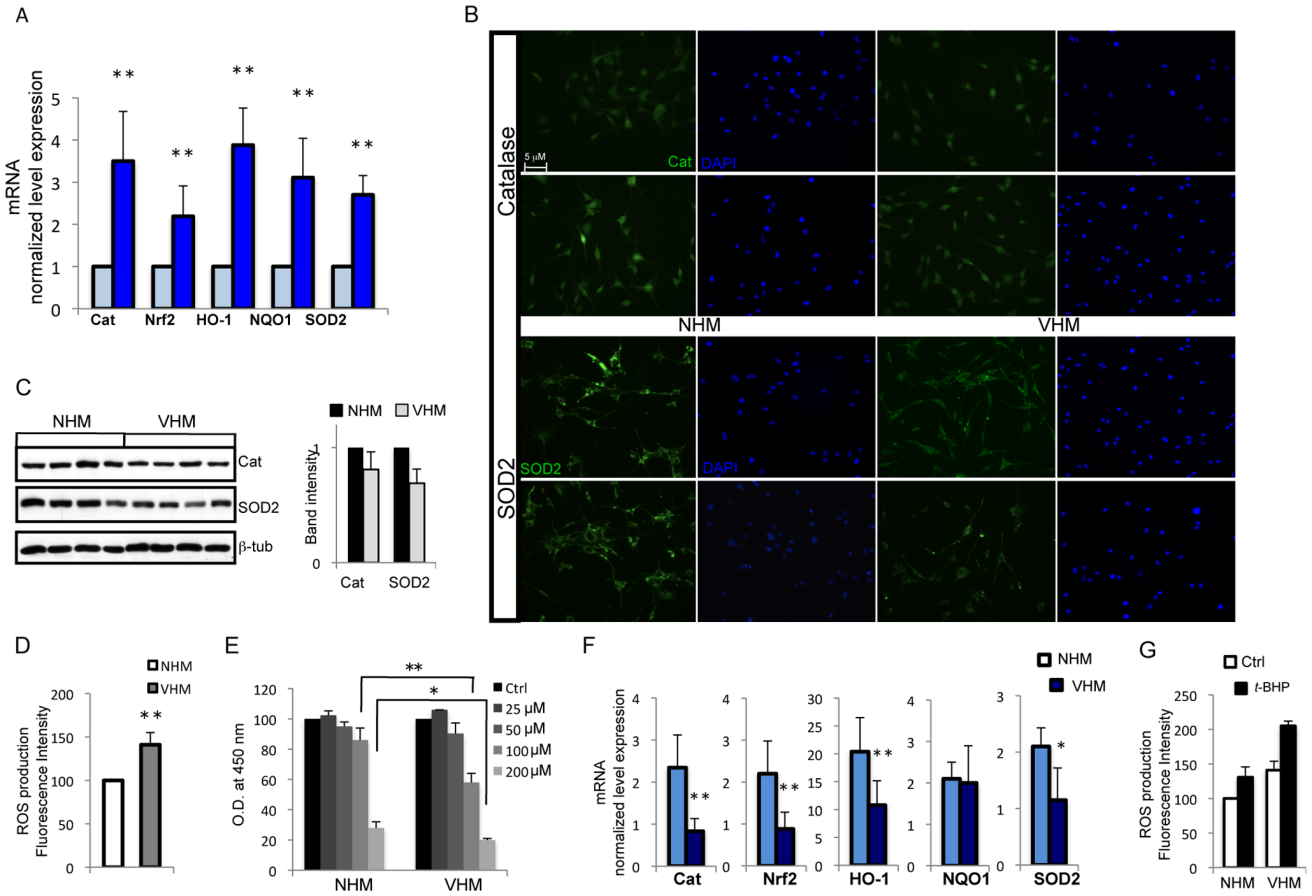
Evidence for constitutive activation of cytoprotective enzymes network in vitiligo melanocyte cultures. (A) Total RNA was extracted from eleven vitiligo and fifteen normal melanocyte cell cultures. RT-PCR analysis was performed for each sample in triplicate. The median ΔC_t_ value, calculated as the differences between the C_t_ value for the gene of interest and that for the endogenous control β-actin was used to calculate 2^−ΔΔCt^, where ΔΔC_t_ represents ΔC_t control_ -ΔC_t vitiligo_. Values represent the means ± SD of ΔΔC_t_. (B) Representative immunoflrurescence reactivity of catalase (upper panel) and SOD2 (lower panel) antibodies in vitiligo (right panel) and normal melanocytes (left panel). Nuclei were labelled with bisbenzidine (DAPI). Original magnification 20x. There are no significant differences between VHM and NHM. (C) One representative western blot and denitometric analysis (NHM n = 9; VHM n = 9) of SOD2 and catalase expression. (D) Measurement of intracellular ROS production in lives cells performed by H_2_DCFDA method. Fluorescence intensity was measured by FACS analysis in duplicate. Data are expressed as –fold increase (VHM n = 7) over the control (NHM n = 8) and represent means ± SD. ** p≤0.01 *versus* control. (E) Comparative analysis of VHM (n = 8) and NHM (n = 8) sensitivity to different doses of *t*-BHP following 24 h treatments. After incubation period cytotoxicity was measured by MTT colorimetric assay. Values reported as O.D. decrease over untreated control represent the means ± SD of experiments performed in triplicate. (F) RT-PCR analysis of detoxifing and antioxidant genes expression. Cells were treated with 100 µM *t*-BHP (or not) for 24 h before RNA extraction. The ΔΔC_t_ value was calculated as reported in (A), VHM (n = 7) and NHM (n = 7). Control values (untreated VHM and NHM) taken as 1, is omitted. The stimulation of Cat, Nrf2, HO-1 and SOD2 mRNA was significantly lower in vitiligo cells whereas NQO1 induction was similar. * p≤0.05; ** p≤0.01 *versus* control.

Previous clinical and experimental observations indicated oxidative stress as a possible biological basis of melanocytes disappearance [3], [12]. In melanocytes isolated from vitiligo patients we observed chronic activation of Nrf2, manganese superoxide dismutase 2 (SOD2), hemeoxigenase-1 (HO-1), NAD(P)H deydrogenase quinone-1 (NQO1) and catalase genes expression that indicated a compromised redox homeostasis (Fig. 1A). The expression of SOD2 and catalase were also investigated at the protein level by immunofluorescence analysis and densitometric evaluation of western blots. At the protein level vitiligo and healthy melanocytes showed similar staining intensity for catalase, whereas SOD2 labeling was slightly lower in vitiligo cells (Fig 1 B and C). A significant increase in ROS concentration (Fig. 1D) could explain the persistent stimulation of antioxidant and detoxification genes in vitiligo melanocytes, whereas attenuated protein expression could be directly caused by altered intracellular redox balance affecting enzymes stability. In fact, it has been demonstrated that acceleration of catalase degradation caused by H_2_O_2_ determines the increase of the corresponding gene transcription, producing a constant stady-state level of protein expression [27]. Activation of antioxidant gene expression indicated a compromised redox homeostasis in melanocytes isolated from vitiligo patients. We decided to further investigate this by studying the effect of induced oxidative stress by tert-butyl-hydroperoxide (*t*-BHP), a membrane-permeant oxidant. As a consequence of peroxide-mediated cell damage a dose-dependent decrease in the number of viable melanocytes was evident (Fig. 1E). At highest *t*-BHP concentrations (100 and 200 µM) the reduction of cell viability was significantly greater in vitiligo human melanocytes (VHM) than in normal human melanocytes (NHM). The dose of 100 µM was selected for further experiments in order to maximize the differences between normal and vitiligo cultures. Consistent with the results obtained from MTT assays, we observed a significant difference in the induction of catalase and detoxification enzyme expression upon exposure of cells to oxidative stress, with the exception of NQO1, which was similar in all cell cultures (Fig. 1F). Consequently, vitiligo cells were not fully competent to counteract the increase of oxygen species (Fig. 1G).

### Alteration of intracellular signaling pathways in vitiligo melanocytes

**Figure 2:**
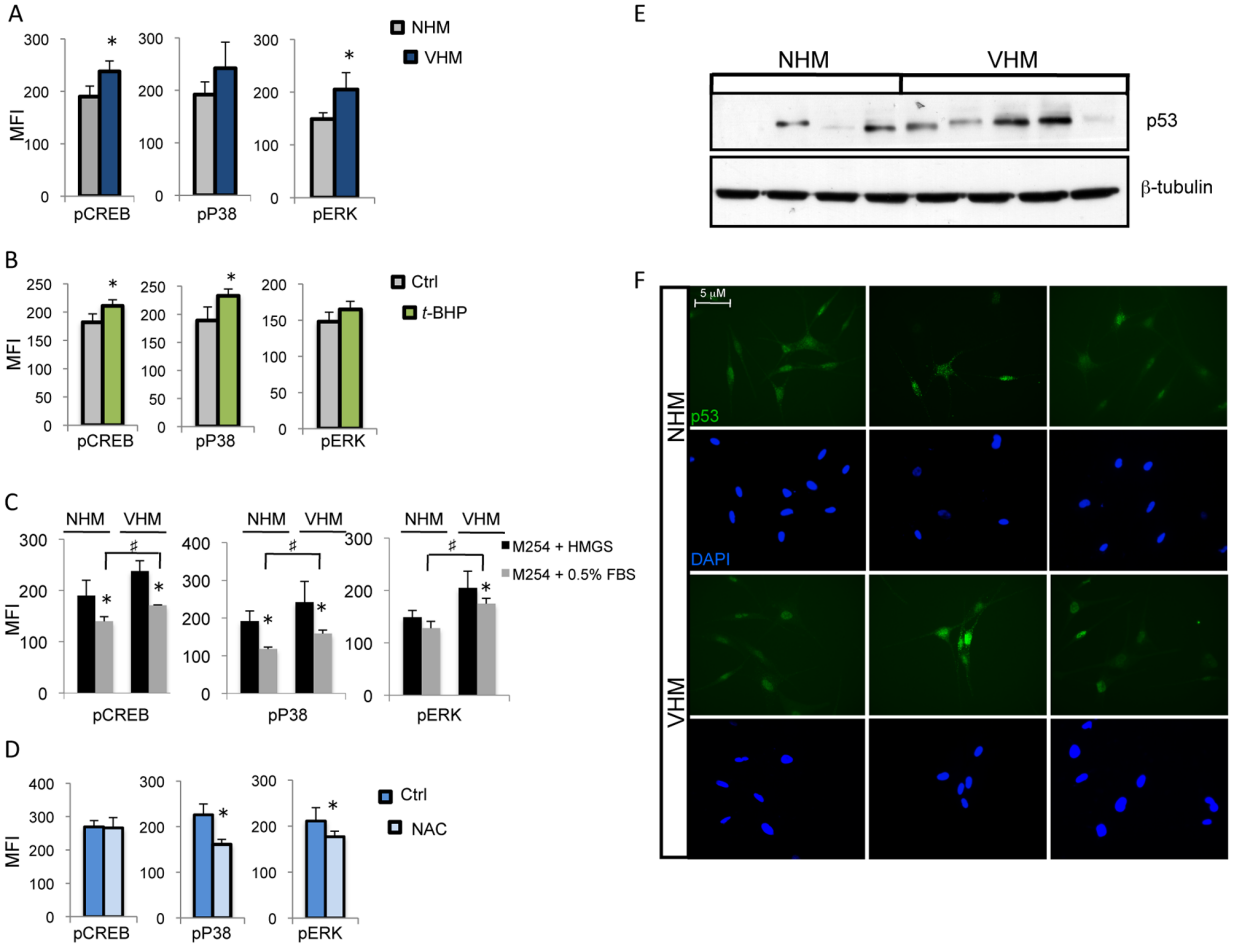
Analysis of MAPK and CREB activating phosphorylation. (A) Comparative analysis of CREB and MAPK level of phosphorylation in VHM (n = 10) and NHM (n = 10) was performed in duplicates by FACS analysis. Histogram represents means ± SD of median fluorescence intensity MFI. (B) NHM (n = 8) demonstrated the same kinase activation profile as VHM (also compare to panel “A”) in response to treatment with *t*-BHP (100 µM). (C) The same immunoreactivities as in (A) were also tested following 24 h of mitogenic factors starvation (M254 medium containing 0.5% FBS) (VHM n = 8; NHM n = 8); NHM and VHM significantly decreased the level of CREB and MAPK phosphorylation. In VHM a significant much higher level of MAPK phosphorylation (compared to NHM) was retained independently to the presence of mitogenic stimulation. p≤0.05 (D) MAPK and CREB level of phosphorylation before and after treatment with NAC (5 mM) for 24 h, VHM = 6 (E) One representative western blot analysis of p53 expression. (F) Immunofluorescence staining demonstrating increased p53 nuclear localization. Representative images of VHM and NHM stained with mouse anti-p53 antibody and DAPI. Original magnification 40x. ♯ and *p≤0.05.

Oxidants can trigger the activation of multiple signaling pathways, including MAPK and CREB. MAPK kinase phosphorylation was found to be dysregulated in vitiligo cells compared to normal melanocytes (Fig. 2A), and CREB, a direct regulator of antioxidant gene expression [28]–[29], was also hyperphosphorylated. The role of oxidative stress in the increased kinases phosphorylation was supported by the demonstration of the same hyperactivated profile in NHM in response to *t*-BHP (Fig. 2B). In addition, vitiligo melanocytes conserved their intrinsic hyperphosphorylation status independently to the presence of mitogens confirming an endogenous source of MAPK activation, such as excessive intracellular ROS levels (Fig 2C). To further confirm the notion that MAPK activation in vitiligo cells is through generation of ROS, *N*-acetyl-L-cystein (NAC), a ROS scavanger, was included in the basal medium. NAC decreased p38 and ERK phosphorylation, whereas, at least at the concentration used, there were not significant modifications of CREB phosphorylation (Fig. 2D). Collectively, these results indicate that in VHM chronic redox imbalance is responsible of a permanent adaptive MAPK-dependent signaling deregulation. Notably, accumulating evidences implicated both CREB and p38 MAPK constitutive phosphorylation in neurodegenration [30]–[31].

Activated kinases can regulate a number of cellular substrates by phosphorylation, leading to diverse cellular responses. Stress-induced p38 acts as a protein kinase to phosphorylate p53, leading to cell cycle arrest, apoptosis and cell survival. Hence, in agreement with earlier studies [16]–[17], analysis of p53 in vitiligo melanocytes by western blot evidenced an increase of protein expression (Fig. 2E) and more importantly, immunofluorescence analysis demonstrated enhanced nuclear distribution of p53 indicating functional activation (Fig. 2F).

### Altered expression of cell cycle regulators

**Figure 3:**
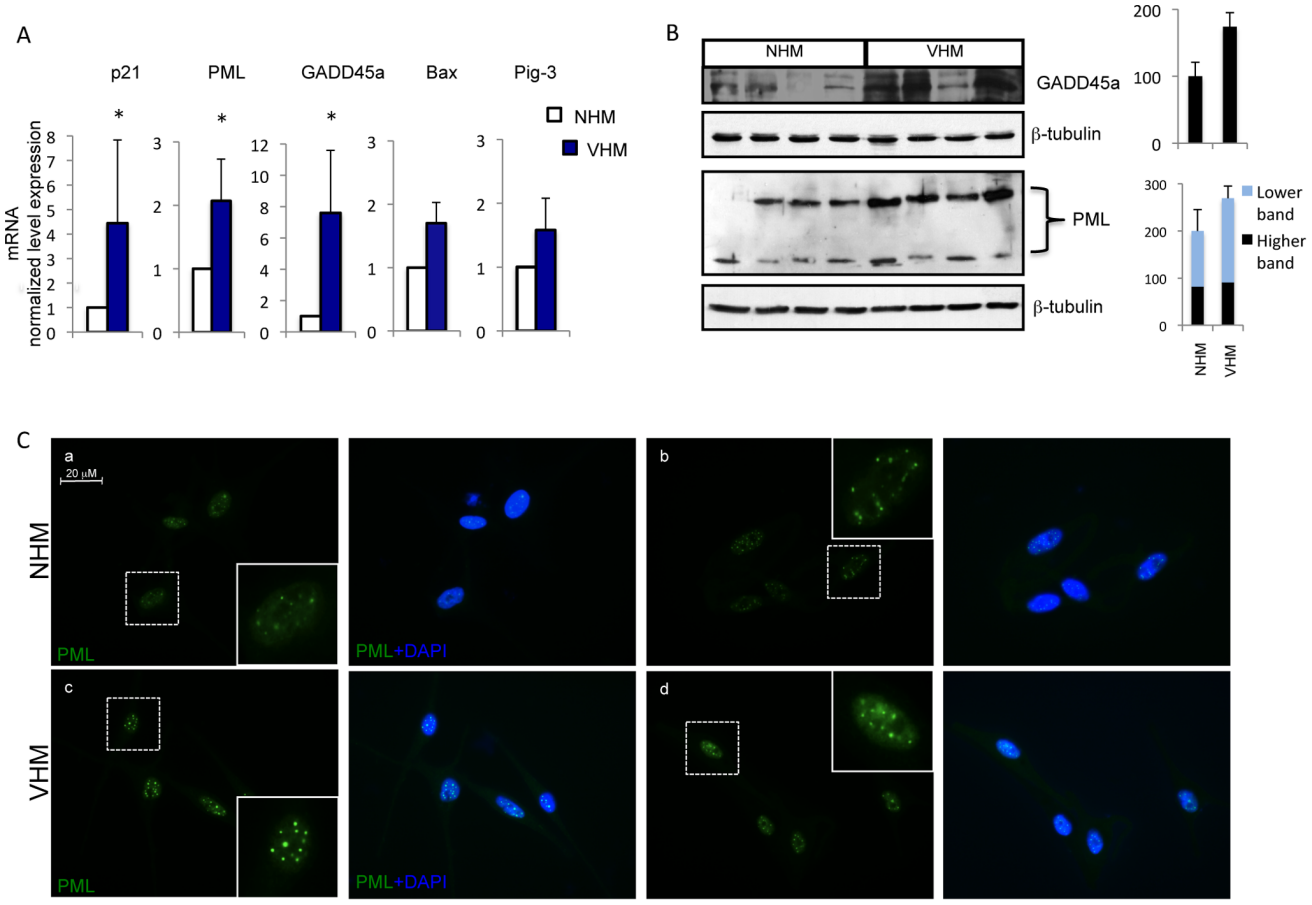
Analysis of p53 target genes expression. (A) Quantitative RT-PCR analysis was performed for each sample in triplicate. The median ΔΔC_t_ value, calculated as reported in Fig.1. Each patient sample (n = 11) was directly compared with all control samples (n = 15) and values represent the means ± SD of fold increase of p53 target genes. (B) The increased expression of GADD45a and PML were also confirmed at protein level by western blot and densitometric analysis (VHM = 9; NHM = 9). For PML two different isoforms were detected and quantified separately. (C) Representative analysis of intracellular distribution of PML by immunofluorescence analysis (VHM = 6; NHM = 6). Nuclei were labelled with bisbenzidine (DAPI). Original magnification 63x. *p≤0.05.

**Figure 4:**
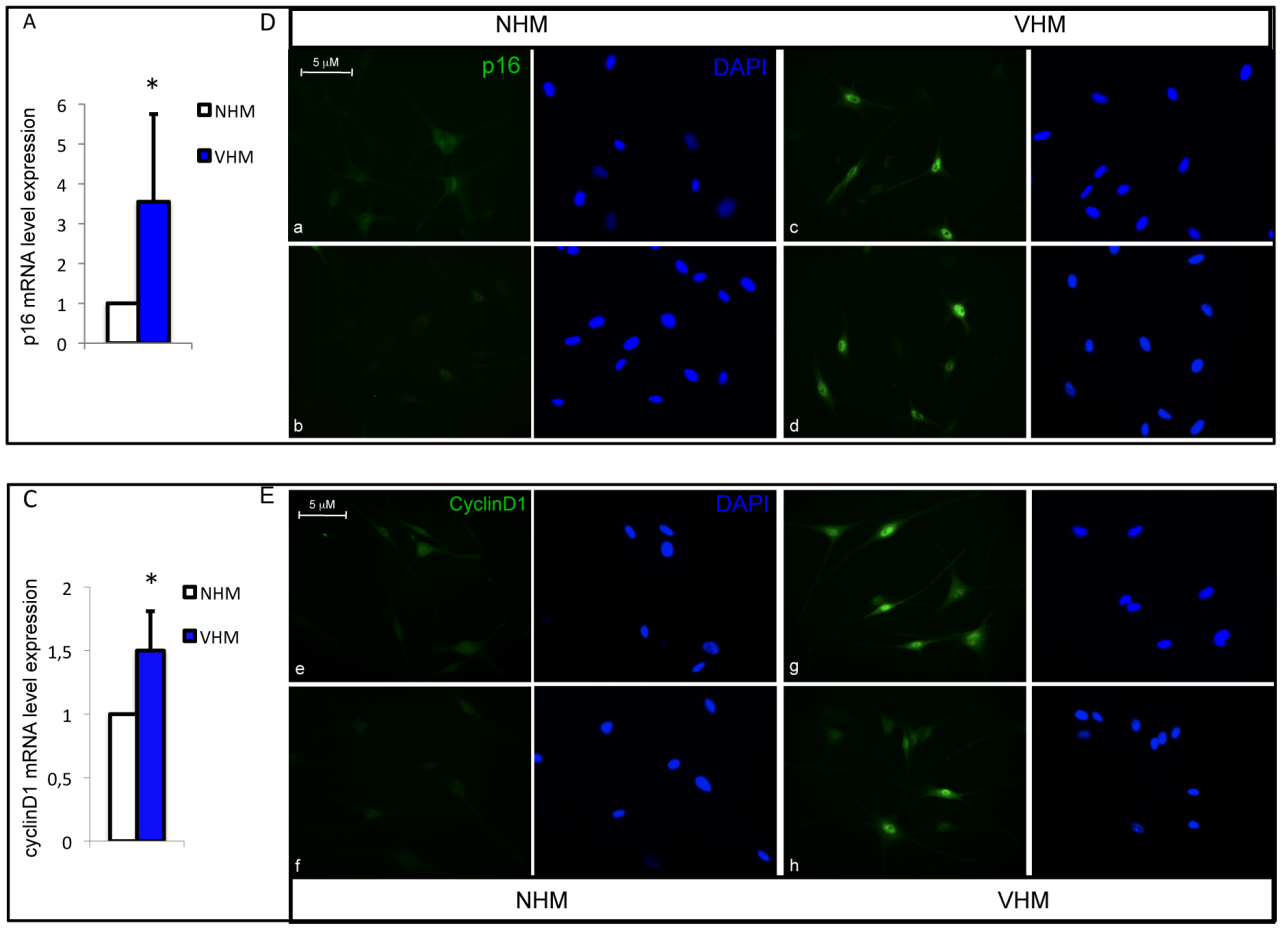
Analysis of cell cycle regulators expression. (A) p16 and (B) CyclinD1 mRNAs (VHM = 11; NHM = 15). (C-D) Immunoflurescence analysis of p16 and cyclinD1 expression and localization. Representative images of VHM (right panel) and NHM (left panel) stained with mouse anti-p16 and anti-cyclinD primary antibodies. Nuclei were labelled with bisbenzidine (DAPI). Original magnification 40x. *p≤0.05.

**Figure 5:**
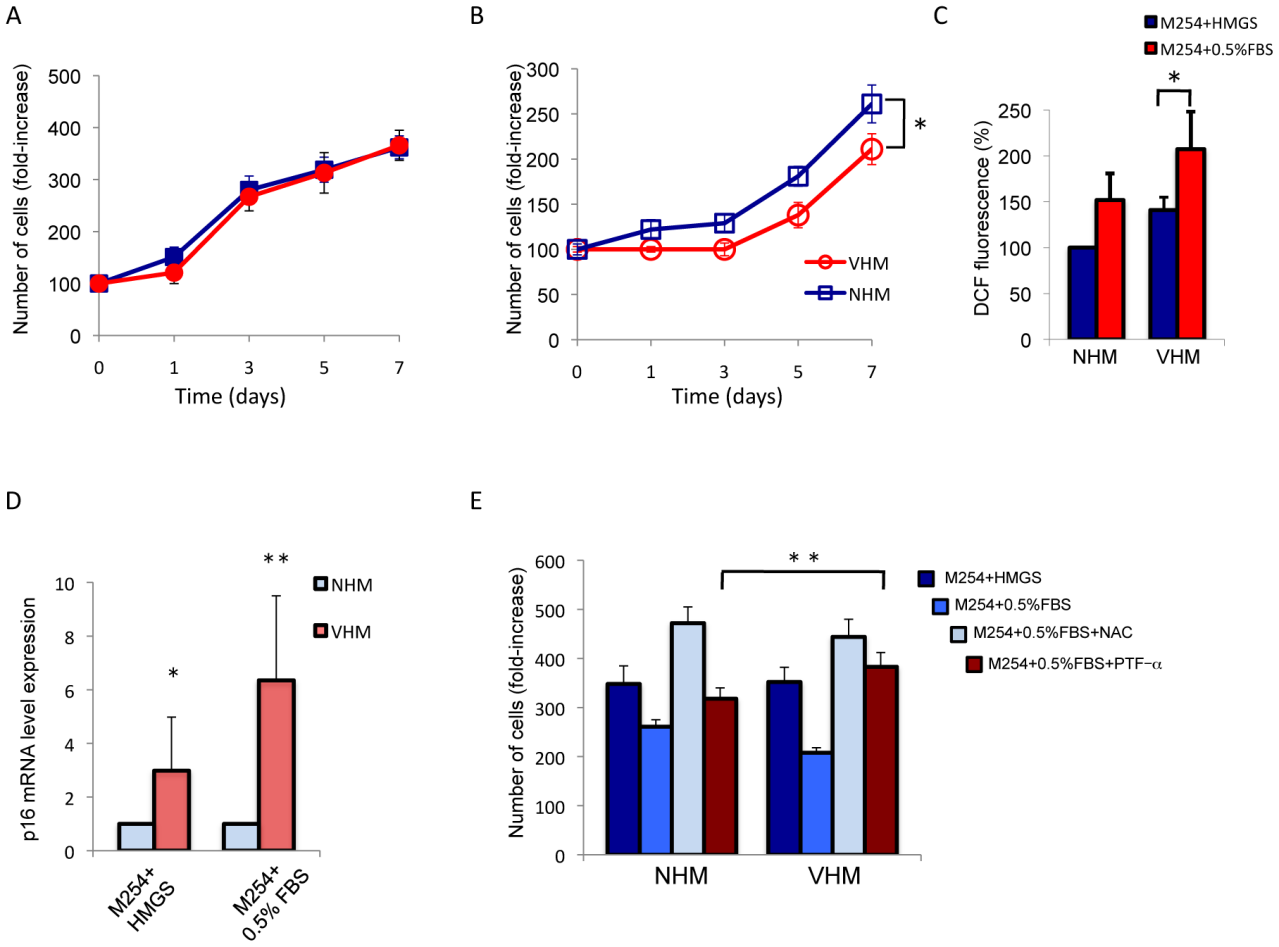
Analysis of cell proliferation. Cells (VHM n = 8; NHM n = 8) were incubated with M254 medium plus HMGS (A) or M254 medium plus 0.5% FBS (basal medium) (B). Growth medium was replaced with fresh medium every 48 h and cells were left to grow for 72, 96 h or a week before Trypan blue exclusion assay. The data show the mean±SD of experiments performed in triplicate. (C) Evaluation of ROS production following 24 h of growth in basal medium. (D) Analysis of mitogen deprivation (24 h) on p16 mRNA in NHM (n = 8) and VHM (n = 8). (E) To investigate the role of intracellular ROS and the specific role of stress-activated p53 on cell proliferation, starved cells were treated with 5 mM *N*-acetyl-L-cystein (NAC) or 5 µM pifithrin-α (PFT-α). A control sample incubated with M254 plus HMGS was also included. After 5 days the number of viable cells were evacuated by Trypan blue exclusion assay. Histograms represent –fold difference ±SD *versus* control (untreated cells at time 0). Experiments were performed in triplicate (NHM n = 6; VHM n = 6). *p≤0.05; **p≤0.01.

Prolonged p53 activation can cause reduced proliferation and/or cell cycle arrest and expression of senescence-related proteins. In vitiligo melanocytes mRNA quantification demonstrated a significant upregulation of several p53 downstream genes involved in cell cycle regulation such as p21, GADD45a (growth arrest and DNA damage-inducible), PML (promyelocytic leukemia) and a slight increase of the expression of genes involved in p53-mediated apoptosis, such as Bax and Pig-3 (Fig. 3A). The expression of the two stress sensors PML and GADD45 was confirmed by western blot analysis (Fig. 3B). Alternative splicing produces a large number of PML isoforms ranging from 97 KDa of PML-I isoform to 48 KDa of PML-VII isoform but only two specific PML splice variants (PML-I and PML-IV) are targeted to the nucleolus by stress conditions [32]–[33]. In adults melanocyte cultures two isoforms were easily detectable: one PML high molecular weight isoform compatible with PML-I and a low molecular weight isoform compatible with PML-VI and PML-VII isoforms. Interestingly, VHM express abundant PML-I isoform that is required for the targeting of PML proteins to the nucleolus and for the formation of very large PML nuclear bodies associated with senescence [33]. Accordingly, immunofluorescence analysis confirmed higher level of PML expression and demonstrated a general increase in PML nuclear bodies size (Fig. 3C). The disequilibrium in cell cycle regulation was also demonstrated by increased expression of p16 in VHM compared to age-matched control (Fig. 4A). Surprisingly, at the same time, we observed abundant expression of the proliferation marker cyclinD1 in vitiligo cells (Fig. 4B). Increased expression of p16 and cyclinD1 was also confirmed by immunofluorescence analysis (Fig. 4C and D). Overall, these data support a possible impairment of cell cycle regulation in vitiligo. In complete culture medium (M254 + HMGS), used for routine growth, no differences were observed between VHM and NHM (Fig. 5A). However, in basal medium (M254+0.5%FBS) vitiligo cells exhibited reduced cell proliferation compared with normal melanocytes (Fig. 5B), suggesting that mitogens starvation impacted the vitiligo cells more than the normal melanocytes. To test whether ROS accumulation plays a role in the proliferation defect observed in VHM during incubation with minimal medium, we measured ROS production under starvation condition. Indeed, upon growth factors starvation, intracellular ROS increased, indicating that cells accumulate peroxides under these conditions (Fig. 5C). Notably, higher levels of intracellular ROS were detected in vitiligo cells compared to controls, also in minimal medium and intracellular, ROS levels reached a more critic concentration in VHM that probably compromised cell proliferation. Moreover, higher difference in the level of p16 mRNA was observed as a consequence to growth factor deprivation (up to 6-fold difference, VHM versus NHM) (Fig. 5D). Results suggest that starvation induces ROS formation intensifying the activation of stress-induced signaling and critical mediator of cellular senescence in vitiligo melanocytes. But, are ROS concentration and stress-dependent signaling important for proliferation control in vitiligo cells? To address this question, we tested the effect of NAC, a general antioxidant and pifithrin-α (PFT-α), a small molecule inhibitor of p53, on growth rate under starvation condition. Addition of either NAC or PFT-α significantly relieved the effect of growth factors deprivation on both normal and vitiligo cells (Fig. 5E). At the end-point NAC caused in VHM and NHM a similar inhibition of starvation-induced growth arrest. Interestingly, the reversibility of growth inhibition obtained by p53 inhibition was significantly higher in vitiligo cells suggesting that p53 overexpression exerts a central role in vitiligo cell cycle regulation.

### Cellular cholesterol, a marker of oxidative stress-induced premature senescence, is increased in vitiligo melanocytes

**Figure 6:**
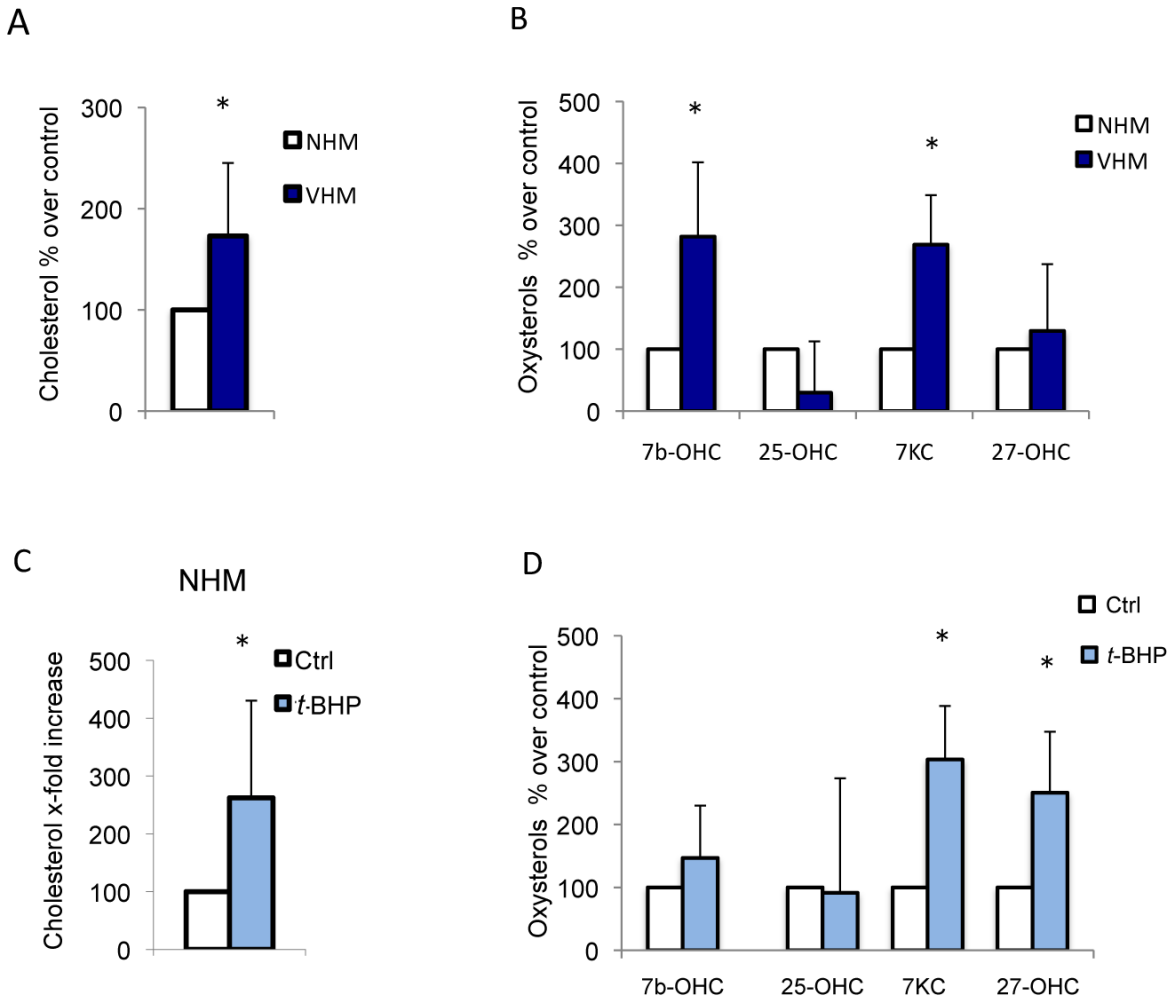
Cholesterol and oxysterols content in VHM and peroxide-treated NHM. (A) The cholesterol and oxysterols (B) levels were measured by GC-MS analysis in VHM (n = 10) and NHM (n = 10). Histogram reports the average ± SD after normalization for protein concentration setting the NHM cholesterol content as 100. (C-D) The cholesterol and oxysterols levels of NHM treated with 100 µM *t*-BHP for 24 h. Results are expressed as percentage ± SD after setting the untreated cells cholesterol content as 100.

Increased cellular cholesterol is associated with replicative senescence *in vitro* as well as with biological aging *in vivo* [34] and may contribute to the onset of degenerative diseases [35]. In VHM cultures the cholelesterol content was increased (Fig. 6A). Furthermore, vitiligo melanocytes showed higher amounts of oxysterols, in particular 7-beta-hydroxycholesterol and 7-ketocholesterol (Fig. 6B). These cholesterol oxidation products have important physiological/pathophysiological roles, including cholesterol homeostasis and oxysterols-induced cell death that can take part in degenerative pathologies [36]. Moreover, the expression of 3-Hydroxy-3-methylglutaryl-coenzyme-A reductase (HMG-CoAR), the rate-limiting enzyme in cholesterol synthesis, was found increased in VHM and in NHM following *t*-BHP treatment (data not shown). Treatment of NHM with *t*-BHP significantly perturbs plasma membrane composition enriching cholesterol and oxysterols content (Fig. 6C and D).

### Authocrine and paracrine activity of vitiligo cells

**Figure 7:**
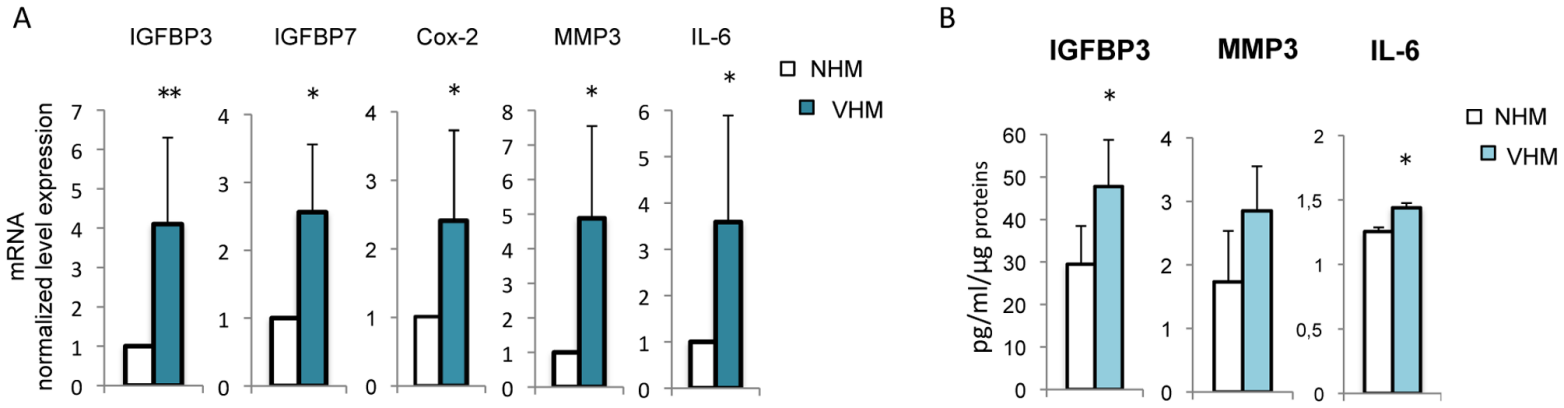
Expression of SASP components. (A) RT-PCR analysis was performed for each sample in triplicate. Each patient sample (n = 10) was directly compared with all control samples (n = 11). (B) Enzyme-linked immunosorbent assay was used to measured intracellular IGFBP3 and IL6 and extracellular MMP3 level of production (NHM n = 8; VHM n = 8). Histogram reports the average ± SD after normalization for protein concentration. *p≤0.05.

In vitiligo melanocytes, chronic oxidative stress appears to activate adaptive program mainly via overexpression of p53 and the activation of the MAPK signaling cascade. Although these processes have been studied in several different cell types, demonstrating a wide association of these markers with decreased proliferation capacity and senescence, it is also known that mechanisms of cellular degeneration and senescence greatly differ between cell types. Permanent damaged human cells secreted many biologically active proteins, a phenotype termed the senescence-associated secretory phenotype (SASP) [37], including cytokines, growth factors and regulators and molecules implicated in cell adhesion and tissue remodeling. In human melanocytes, senescence is associated with an increased production of insulin-like growth factor binding proteins (IGFBP), and treatment with recombinant exogenous IGFBP7 is sufficient to induce senescence in melanocytes and apoptosis in certain melanoma cell lines [38]. Interestingly, the expression of IGFBP3 and IGFBP7 were markedly higher in VHM (Fig. 7A). Notably, *IGFBP3* gene transcription is under the control of p53 [39]. Metalloproteinase-3 (MMP3), a classical marker of senescence *in vitro*, and IL-6 a senescence-associated inflammatory mediator, were highly expressed by VHM at both mRNA (Fig. 7A) and protein level (Fig. 7B). Cyclooxygenase-2 (Cox-2), an inducible enzyme of the prostaglandin biosynthesis pathway, involved in stress-induced senescence [40] was weakly but significantly upregulated.

### Stress and senescent-associated markers validation on vitiligo tissue biopsies

**Figure 8:**
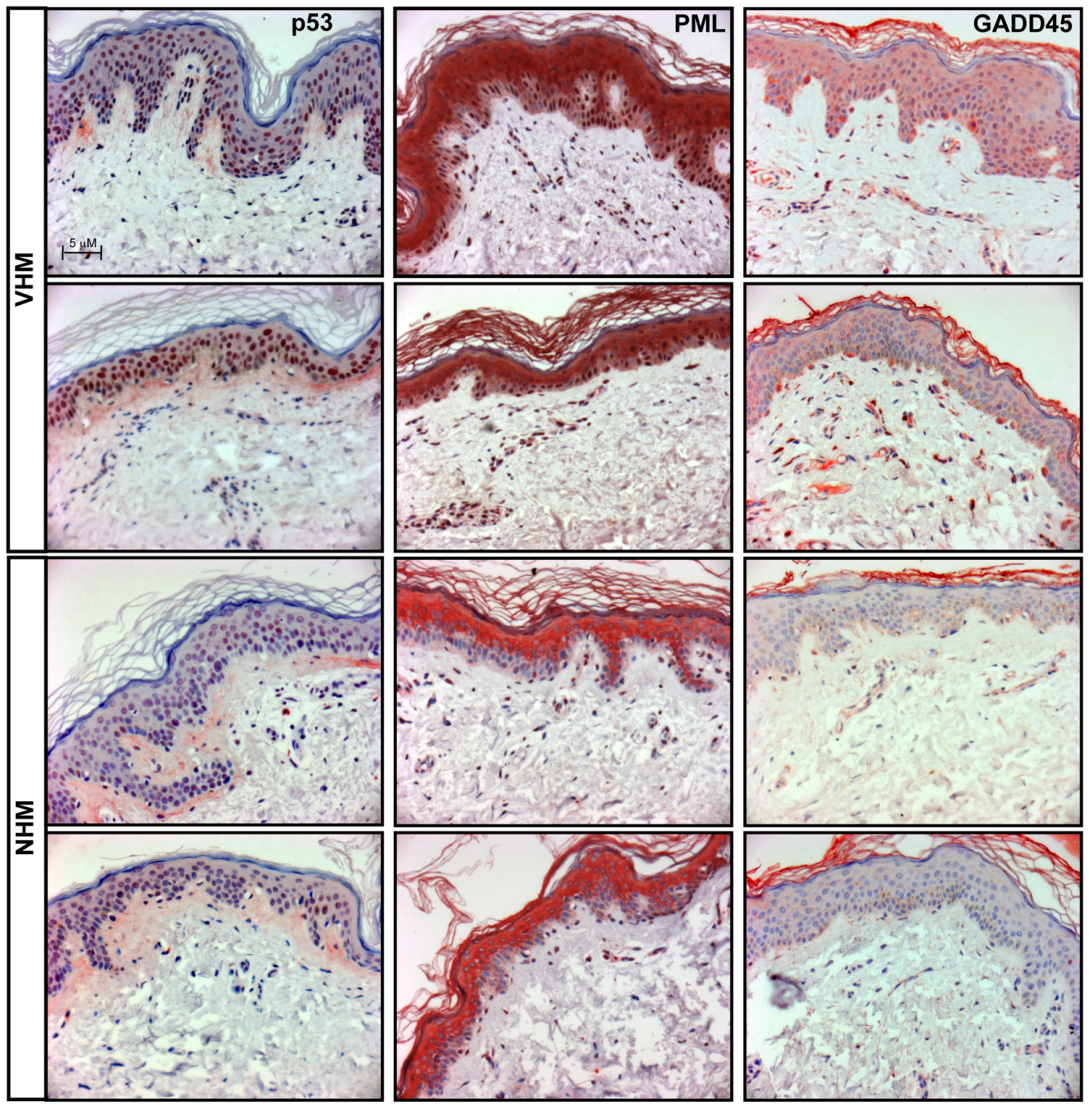
Analysis of stress and senescent-associated markers on vitiligo tissue biopsies. P53 and its senescent-associated target genes PML and GADD45a were analyzed on tissue biopsies (VHM = 4; NHM = 4) by immunohistochemistry.

**Figure 9:**
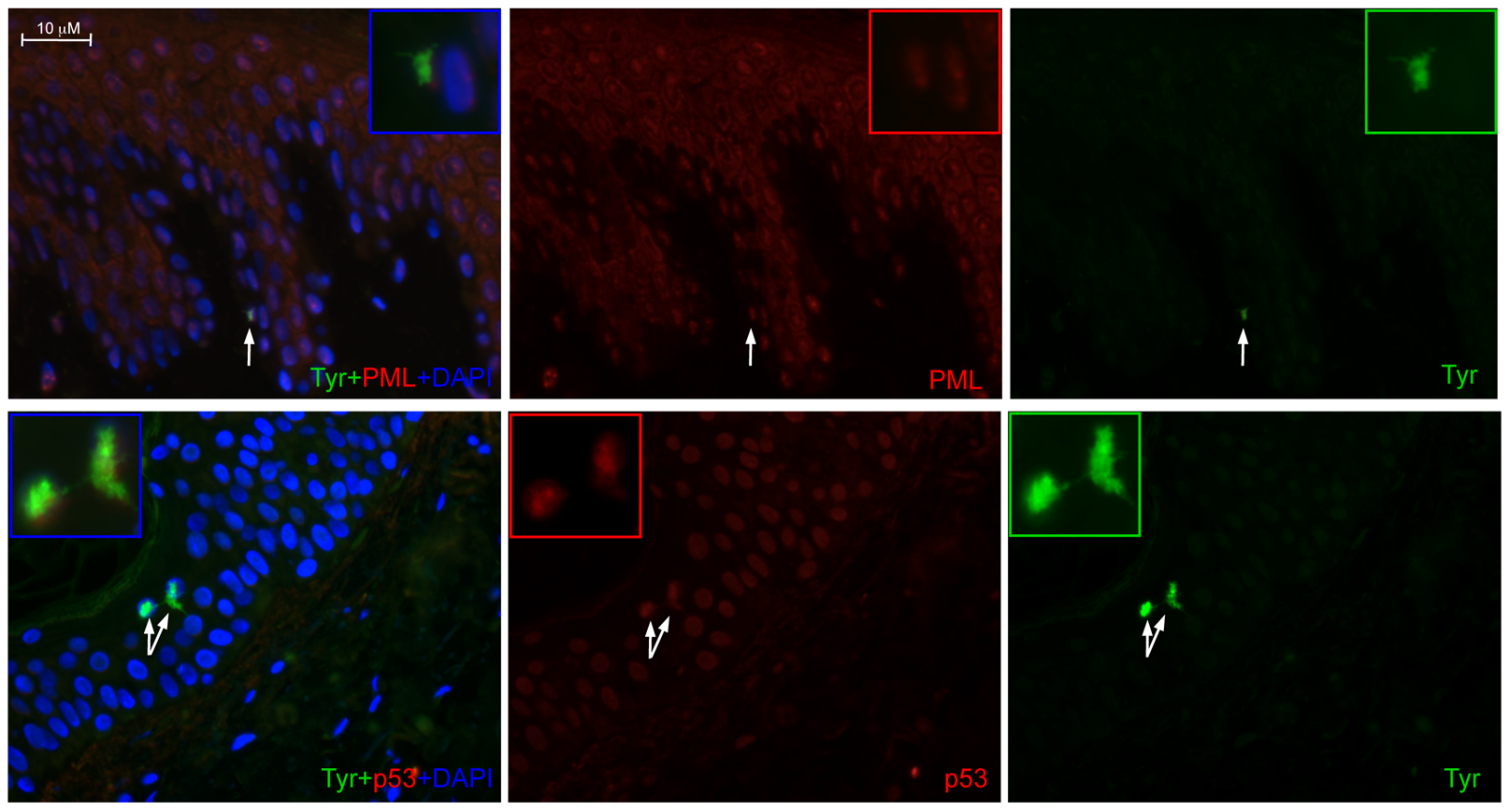
p53 and PML expression co-localize with melanocyte specific markers. Double immunofluoresce staining was used to co-localize the expression of the melanocyte-specific marker tyrosinase and the expression of p53 and PML. Nuclei were labelled with bisbenzidine (DAPI). Original magnification 40×.

Vitiligo melanocyte cultures displayed the alteration of several stress-induced markers including p53-dependent senescent proteins. To confirm *in vitro* results we analyzed in skin biopsies the expression of p53, and of two p53 target genes involved in cell senescence: PML and GADD45 (Fig. 8). P53 localized exclusively in the nuclear compartment in both healthy and vitiligo samples with a stronger signal in vitiligo tissue. In healthy sample PML was weakly detected only in the cytoplasm of upper epidermal layers whereas in vitiligo biopsies a strong citoplasmic signal was associated with an intense nuclear positivity of all epidermal layers. Normal skins were negative for the expression of GADD45 whereas vitiligo samples showed a diffuse citoplasmic staining with some sporadic cell in the basal layer showing an intense positivity. Interestingly, our results demonstrated that the activation of p53-dependent pathway was not restricted to melanocytes but was generally diffuse in all the epidermis. To confirm that not only keratinocytes but also melanocytes overexpress senescent-associated marker we performed double immunofluorescence analysis confirming the co-expression of both the melanocyte specific marker tyrosinase and p53 or PML in the same cell (Fig.9).

## Discussion

Vitiligo is an acquired disorder of skin pigmentation characterized by localized destruction of cutaneous melanocytes that lacks a satisfactory explanation. Mitochondrial dysfuction, oxidative stress, and immune mechanisms [1] are among the factors implicated in melanocytes disappearance in vitiligo. A critical question is why specific melanocytes are selectively vulnerable in vitiligo disease. The skin is one of the most impacted organs by environmental-induced stress and the slow melanocytes turnover [41], compared to keratinocytes, could contribute to the accumulation of damage in growth-arrested cells in vitiligo skin. Moreover, the melanin biosynthetic pathway is a potential additional source of ROS inside the pigment-forming cells. Thus, it is possible that, in some circumstances, melanogenesis primes an acute stress response in vitiligo cells, which are more prone to lose their redox balance equilibrium. Interestingly, increased metabolic stress and failure to detoxify potential toxin due to the presence of neuromelanin has been proposed as a pathogenic mechanism in Parkinson's disease [42].

Several lines of evidence showed relevant biological similitude between vitiligo and neurodegenerative diseases. Interestingly due to common embryonic origin of melanocytes and nervous system neurons, skin-derived melanocytes were already proposed as a possible model system to investigate pathological behaviors of less accessible nervous system neurons [43]. Impaired activity of the mitochondrial electron transport chain complex I in vitiligo cells and increase in the expression of mitochondrial malate dehydrogenase activity argue for an important role of mitochondrial defective functionality in the pathogenesis of vitiligo [3], [13]. Similarly, there are several degenerative disorders with evidence of mitochondrial involvement. These include Parkinson disease (where a complex I defect is described and free radicals are generated from dopamine metabolism [44], amyotrophic later sclerosis, and Alzheimer disease, where there is evidence to suggest mitochondrial involvement perhaps secondary to other abnormalities [38].

Here, we have demonstrated that continuous intracellular ROS generation in the vitiligo melanocytes leads to a constitutive stimulation of antioxidant enzymes expression at the mRNA level. Although the mRNA expression of antioxidant enzymes is increased, the protein levels of catalase and SOD2 remain low, demonstrating an intrinsically higher detoxifying-enzyme turnover that is independent of environmental stresses. An increased level of expression and decreased level of activity of antioxidant enzymes are often observed during a pathologically prolonged state of oxidative stress, such as in Alzheimer's disease [44], and are likely to reflect the demand to increase expression due to attenuated enzyme activity that could be directly caused by the oxidative damage. Our results are consistent with previous studies demonstrating that higher Nrf2-dependent transcriptional activity is required for the maintenance of redox balance in vitiligo skin [15] and that catalase protein expression and activity are low in vitiligo skin [4]. Intriguingly, lowered catalase protein activity, elevated production of H_2_O_2_ and a refractory stress response have been reported to correlate with the aged phenotype of hair follicle melanocytes [45]. ROS escape results in the activation of cytosolic stress pathways including the upregulation of p53 that represents a central integration point for various signaling pathways. P53 functions in a stimulus-dependent and cell-type-dependent manner leading to cell cycle arrest, senescence or apoptosis as extreme outcome. In VHM we observed permanent adaptive changes but cells still function normally, or slightly suboptimally in a pro-oxidant environment. In this situation VHM showed a higher level of expression of senescent-associated p53-target genes such as PML, GADD45, and IGFBP3 suggesting a pre-senescent phenotype. Interestingly, IGFP3 has been also implicated in neuronal degeneration and clinical symptoms in Alzheimer disease [46]. Moreover, VHM demonstrated a significant increase in p16 expression that did not correlate with donor chronologic age. Over a certain threshold (in terms of severity of oxidative stress), the loss of oxidative equilibrium affects cell survival (*t*-BHP) and cell proliferation (mitogens starvation). Importantly, pharmacological inhibition of p53 prevents mitogens deprivation-dependent growth arrest strongly in VHM than NHM demonstrating that p53 overexpression provides a crucial barrier to vitiligo cells proliferation.

An unexpected observation of our study is the increased expression of the proliferation marker cyclinD1 in vitiligo cells. A possible explanation is that, as suggested for Alzheimer neurons, accumulation of cyclinD1 an consequent cell cycle abnormalities represents an abortive entry into the cell cycle [47]. A similar scenario has been proposed in the hypermitogenic cell cycle arrest that is characterized by high level of cyclinD1 [48]. Classical growth arrest caused by growth factor withdrawal is essentially an exit from the cell cycle characterized by the inactivation of both upstream (MAPK signaling) and downstream proliferation factors (cyclins). Hypermitogenic cell cycle arrest occurs when mitogen-activate pathways are active but cell progression is blocked downstream (for example p16 over-expression), and a conflicting signal is generated leading to a senescence-like hypermitotic arrest. The integration of upstream and downstream signaling in resting cells could lead to slow cell death/senescence. Most of the epidermal melanocytes are in a resting state and it is possible that prolonged production of inflammatory hormones in vitiligo results in a dangerous activation of melanocytes as described in the Koebner's phenomenon [49]. According with the concept that inappropriate activation of mitogen-activated and growth-promoting pathways may drive senescence, hypermitogenic type of senescence is a form of an altered growth and not necessarily an absence of cell cycle proliferation [50]. Increased cellular functions (hypertrophy, pro-inflammatory and hyper-secretory phenotypes), resembling senescence caused by DNA damaging agents, is the most important marker of hypermitogenic arrest [51]. From the medical point of view, these hyper-functions contribute to age-related diseases, such as atherosclerosis, Alzheimer's and other neurodegenerative diseases and macular degeneration [52]. In the case of vitiligo cells, long-term exposure to subcytotoxic oxidative stress could cause chronic over-stimulation of MAPK, activation of p53, and induction of both p16 and cyclinD1 expression at the same time, resembling pro-senescent hypermitotic phenotype. A progressive rise of oxidative stress and impaired ability to cope with stressful stimuli may not be exclusive to epidermal melanocytes in vitiligo patients. Like melanocytes, peripheral blood mononuclear cells [13]–[14], cultured skin keratinocytes [53], and fibroblasts from vitiligo patients show markers of oxidative stress and increases in cholesterol content (unpublished data). Interestingly, similar systemic alterations of cholesterol homeostasis have also been reported in Alzheimer's disease [19], [54]. According with the non-exclusive involvement of melanocytes, a previous study reported a shorter in vitro life span and senescence markers in keratinocytes cultured from involved skin in vitiligo patients [55]. In this study immunohistochemical analysis of skin biopsies also showed that the activation of p53-dependent signaling is not restricted to melanocytes but also involve keratinocytes. The acquisition of senescence-associated secretory phenotypes, combing the effect of intracellular signaling pathway deregulation with extracellular environments perturbation, could affect the behavior of neighboring cells and also systemic homeostasis since melanocytes could release into circulation melanocyte signaling molecules and neurotransmitter-like factors [56]. The senescent-prone phenotype could also provide a molecular explanation to the observation that vitiligo is associated with fewer risks of skin cancer [57]–[58] since premalignant human nevi contain cells that express senescence markers [59].

Histological data have provided good evidence of immune-inflammatory components in the upper dermis and the dermal-epidermal interface of vitiligo lesional and peri-lesional skin [7]. Intriguingly, repeated stimulations of fibroblasts with tumor necrosis factor-alpha (TNF-α) or IL-α, two pro-inflammatory cytokines that are higher expressed in lesional skin of vitiligo patients [38], [60], stimulate the appearance of biomarkers of *in vitro* aging in a stress-dependent manner [61]. This example of long-term effects of cytokines on human fibroblasts, and potentially on other cell types, suggests that paracrine-acting messengers derived from skin immune system and keratinocytes may be a focus responsible for the start of melanocytes premature senescence and vitiligo pathological process.

The senescent-prone melanocytes not only explain the disappearance of functional melanocytes in lesional epidermis but also the loss of normal regeneration that could be caused by depletion of stem or progenitor cells. Interestingly, hyperactivation of p53 have been implicated in age-associated decline in tissue stem cell regenerative function [62] and degenerative disorders [63]. This depletion will compromise tissue repair, regeneration and normal turnover, leading to functional decrements. Moreover, the *in vitro* melanocytes cell culture model could represent a readily accessible human cell model for the development of new therapy strategies capable of inhibiting degeneration in the vitiligo cells and other degenerative diseases.

## Supporting Information

Table S1 **List of primers used for quantitative real time PCR.** Sequences of primers indicated with an F correspond to sense strands and with an R correspond to anti-sense.
(TIF)

## References

[1] Taieb A, Picardo M (2009) Clinical practice: Vitiligo. N Engl J Med 360: 160–169. 19129529 10.1056/NEJMcp0804388

[2] Le Poole IC, Luiten RM (2008) Autoimmune etiology of generalized vitiligo. Curr Dir Autoimmun 2008 10: 227–243. 10.1159/000131485 18460889

[3] Dell'Anna ML, Ottaviani M, Albanesi V, Vidolin AP, Leone G, et al (2007) Membrane lipid alterations as a possible basis for melanocyte dagenaration in vitiligo. J Invest Dermatol 127: 1226–1233. 17235326 10.1038/sj.jid.5700700

[4] Schallreuter KU, Rübsam K, Gibbons NC, Maitland DJ, Chavan B, et al (2008) Methionine sulfoxide reductases A and B deactivated by hydrogen peroxide (H2O2) in the epidermis of patients with vitiligo. J Invest Dermatol 128: 808–815. 17943184 10.1038/sj.jid.5701100

[5] Gauthier Y, Cario Andre M, Taϊeb A (2003) A critical appraisal of vitiligo etiologic theories. Is melanocyte loss a melanocytorrhagy? Pigment Cell Res 16: 322–332. 12859615 10.1034/j.1600-0749.2003.00070.x

[6] Spritz RA (2008) The genetics of generalized vitiligo. Curr Dir Autoimmun 10: 244–257. 18460890 10.1159/000131501

[7] Taieb A (2011) Vitiligo as an inflammatory skin disorder: a therapeutic perspective. Pigment Cell Mel Res 25: 9–13. 10.1111/j.1755-148X.2011.00939.x 22099450

[8] Medrano EE, Nordlund JJ (1990) Successful culture of adult human melanocytes obtained from normal and vitiligo donors. J Invest Dermatol 95: 441–445. 1698887

[9] Dell'Anna ML, Cario-André M, Bellei B, Taieb A, Picardo M (2012) In vitro research on vitiligo: strategies, principles, methodological options, and common pitfalls. Exp Dermatol 21: 490–496. 22716243 10.1111/j.1600-0625.2012.01506.x

[10] Maresca V, Roccella M, Rocella F, Camera E, Del Porto G, et al (1997) Increased sensivity to peroxidative agents as a possible pathogenic factor of melanocytes damage in vitiligo. J Invest Dermatol 109: 310–313. 9284096 10.1111/1523-1747.ep12335801

[11] Boissy RE, Manga P (2004) On the etiology of contact/occupational vitiligo. Pigment Cell Res 17: 208–214. 15140065 10.1111/j.1600-0749.2004.00130.x

[12] Schallreuter KU, Moore J, Wood JM, Beazley WD, Gaze DC, et al (1999) In vivo and in vitro evidence for hydrogen peroxide (H2O2) accumulation in the epidermis of patients with vitiligo and its successful removal by a UVB-activated pseudocatalase. J Invetsig Dermatol Symp Proc 4: 91–96. 10.1038/sj.jidsp.5640189 10537016

[13] Dell'Anna ML, Urbanelli S, Mastrofrancesco A, Camera E, Iacovelli P, et al (2003) Alterations of mitochondria in peripheral blood monoculear cells of vitiligo patients. Pigment Cell Res 16: 553–559. 12950736 10.1034/j.1600-0749.2003.00087.x

[14] Giovannelli L, Bellandi S, Pitozzi V, Fabbri P, Dolara P, et al (2004) Increased oxidative DNA damage in mononuclear leukocytes in vitiligo. Mutat Res 556: 101–106. 15491637 10.1016/j.mrfmmm.2004.07.005

[15] Natarajan VT, Singh A, Kumar AA, Sharma P, Kar HK, et al (2010) Transcriptional upregulation of Nrf2-dependent phase II detoxification genes in the involved epidermis of vitiligo vulgaris. J Invest Dermatol 130: 2781–2789. 20664557 10.1038/jid.2010.201

[16] Schallreuter KU, Behrens-Williams S, Khalig TP, Picksley SM, Peters EM, et al (2003) Increased epidermal functioning wild-type p53 expression in vitiligo. Exp Dermitol 12: 268–277. 10.1034/j.1600-0625.2003.00084.x 12823440

[17] Salem MM, Shalbaf M, Gibbons NC, Chavan B, Thornton JM, et al (2009) Enhanced DNA binding capacity on up-regulated epidermal wild-type p53 in vitiligo by H2O2-mediated oxidation: a possible repair mechanism for DNA damage. FASEB J 23: 3790–380. 19641144 10.1096/fj.09-132621

[18] Schallreuter KU, Bahadoran P, Picardo M (2008) Vitiligo pathogenesis: autoimmune disease, genetic defect, excessive reactive oxygen species, calcium imbalance, or what else? Exp Dermatol 17: 139–140. 18205713 10.1111/j.1600-0625.2007.00666_1.x

[19] Jimbow K, Roth SI, Fitzpatrick TB, Szabo G (1975) Mitotic activity in non-neoplastic melanocytes in vivo as determined by histochemical, autoradiographic, and electron microscope studies. J Cell Biol 66: 663–670. 808553 10.1083/jcb.66.3.663 PMC2109461

[20] Pani A, Dessi S, Diaz G, La Colla P, Abete C, et al (2009) Altered cholesterol ester cycle in skin fibroblasts from patients with Alzheimer's disease. J Alzheimers Dis 18: 829– 841. 19749436 10.3233/JAD-2009-1193

[21] Schapira AH, Cooper JM, Dexter D, Clark JB, Jenner B, et al (1990) Mitochondrial complex I deficiency in Parkinson's disease. J Neurochem 54: 823– 827. 2154550 10.1111/j.1471-4159.1990.tb02325.x

[22] Mendonsa G, Dobrowolska J, Lin A (2009) Molecular profiling reveals diversity of stress signal transduction cascades in highly penetrant Alzheimer's disease human skin fibroblasts. PLoS One 4: e4655. 19247475 10.1371/journal.pone.0004655 PMC2644820

[23] Lee HC, Wei YH (2001) Mitochondrial alterations, cellular response to oxidative stress and defective degradation of proteins in aging. Biogerentology 2: 231–244. 10.1023/a:1013270512172 11868898

[24] Chen J, Goligorsky MS (2006) Premature senescence of endothelial cells: Methusaleh's dilemma. *Am* J Physiol Heart Circ Physiol 290: 1729–1739. 10.1152/ajpheart.01103.2005 16603702

[25] Esiri MM (2007) Ageing and the brain. J Pathol 211: 181–187. 17200950 10.1002/path.2089

[26] Tobin DJ, Swanson NN, Pittelkow MR, Peters EM, Schallreuter KU (2000) Melanocytes are not absent in lesional skin of long duration vitiligo. J Pathol 191: 407–416. 10918216 10.1002/1096-9896(2000)9999:9999<::AID-PATH659>3.0.CO;2-D

[27] Eising R, Süselbeck B (2003) Turnover of catalase heme and apoprotein moieties in cotyledons of sunflowers seedings. Plant Physiol 97: 1422–1429. 10.1104/pp.97.4.1422 PMC1081181 16668566

[28] Bedogni B, Pani G, Colavitti R, Riccio A, Borrello S, et al (2003) Redox regulation of cAMP-responsive element–binding protein and induction of manganous superoxide dismutase in nerve growth factor-dependent cell survial. J Biol Chem 278: 16510–16519. 12609977 10.1074/jbc.M301089200

[29] Krönke G, Bochkov VN, Huber J, Gruber F, Blüml, et al (2003) Oxidized phospholipids induce expression of human heme oxygenase-1 involving activation of cAMP-responsive element-binding protein. J Biol Chem 278: 51006–51014. 14523007 10.1074/jbc.M304103200

[30] Munoz L, Ammit AJ (2010) Targeting p38 MAPK pathway for the treatment of Alzheimer's disease. Neuropharmacology 58: 561–568. 19951717 10.1016/j.neuropharm.2009.11.010

[31] Müller M, Cárdenas C, Mei L, Cheung KH, Foskett JK (2011) Costitutive camp response element binding protein (CREB) activation by Alzheimer's disease presenilin-driven inositol trisphosphate receptor (InsP3R) Ca2+ signaling. Proc Natl Acad Sci USA 108: 13293–13298. 21784978 10.1073/pnas.1109297108 PMC3156223

[32] Jensen K, Shiels C, Freemont PS (2001) PML protein isoforms and RBCC/TRIM motif. Oncogene 20: 7223–7233. 11704850 10.1038/sj.onc.1204765

[33] Condemine W, Takahashi Y, Le Bras M, de Thé H (2007) A nucleolar targeting signal in PML-I addresses PML to nucleolar caps in stressed or senescent cells. J Cell Sci 120: 3219–3927. 17878236 10.1242/jcs.007492

[34] Nakamura M (2003) Kondo H, Shimada Y, Waheed AA, Ohno-Iwashita Y (2003) Cellular aging-dependent decrease in cholesterol in membrane microdomains of human diploid fibroblasts. Exp Cell Res 290: 381–390. 14567995 10.1016/s0014-4827(03)00343-4

[35] Ohno-Iwashita Y, Shimada Y, Hayashi M, Inomata M (2010) Plasma membrane microdomains in aging and disease. Getriatr Gerontol Int 10: 41–52. 10.1111/j.1447-0594.2010.00600.x 20590841

[36] Gamba P, Leonarduzzi G, Tamagno E, Guglielmotto M, Testa G, et al (2011) Interaction between 24-hydroxycholesterol, oxidative stress, and amyloid-b in amplifying neuronal damage in Alzheimer's disease: three partners in crime. Aging Cell 10: 403–417. 21272192 10.1111/j.1474-9726.2011.00681.x

[37] Coppé JP, Patil F, Rodier F, Sun Y, Muñoz DP, et al (2008) Senecence-associated secretory phenotypes reveal cell-nonautonomous functions of oncogenic RAS and the p53 tumor suppressor. PLoS Biol 6: 2853–2868. 19053174 10.1371/journal.pbio.0060301 PMC2592359

[38] Wajapeyee N, Serra RW, Zhu X, Mahalingam M, Green MR (2008) Oncogenic BRAF induces senescence and apoptosis through pathways mediated by the secreted protein IGFBP7. Cell 132: 363–374. 18267069 10.1016/j.cell.2007.12.032 PMC2266096

[39] Kim KS, Kim MS, Seu YB, Chung HY, Kim JH, et al (2007) Regulation of replicative senescence by insulin-like growth factor-binding protein 3 in human umbilical vein endothelial cells. Aging Cell 6: 535–545. 17635417 10.1111/j.1474-9726.2007.00315.x

[40] Zdanov S, Bernard D, Debacq-Chainiaux F, Martien S, Gosselin K, et al (2007) Normal or stress-induced fibroblast senescence involves COX-2 activity. Exp Cell Res 313: 3046–3056. 17560572 10.1016/j.yexcr.2007.04.033

[41] Yaar M, Gilchrest 1BA (2001) Ageing and photoageing of keratinocytes and melanocytes. Clin Exp Dermatol 26: 583–591. 11696062 10.1046/j.1365-2230.2001.00895.x

[42] Lang AE, Lozano AM (1998) Parkinson's disease. First of two parts. N Engl J 339: 1130–1143. 10.1056/NEJM199810153391607 9770561

[43] Yaar M, Park HY (2011) Melanocytes a window into the nervous system. J Invest Dermatol 132: 835–845. 22158549 10.1038/jid.2011.386

[44] Akterin S, Cowburn RF, Miranda-Vizuete A, Jimenez A, Bogdanovic N, et al (2006) Involvement of glutaredoxin-1 and thioredoxin-1 in beta-amyloid toxicity and Alzheimer's disease. Cell Death Differ 13: 1454–1465. 16311508 10.1038/sj.cdd.4401818

[45] Kauser S (2011) Westgate GE, Green MR, Tobin DJ (2011) Human hair follicle and epidermal melanocytes exhibit striking differences in their aging profile which involves catalase. J Invest Dermatol 131: 979–982. 21191398 10.1038/jid.2010.397

[46] Ikonen M, Liu B, Hashimoto Y (2003) Interaction between the Alzheimer's survival peptide humanin and insulin-like growth factor-binding protein 3 regulates cell survival and apoptosis. Proc Natl Acad Sci USA 100: 13042–13047. 14561895 10.1073/pnas.2135111100 PMC240741

[47] Malik B, Currais A, Andres A (2008) Loss of neuronal cell cycle control as a mechanism of neurodegeneration in the presenilin-1 Alzheimer's disease brain. Cell Cycle 7: 637–646. 18239458 10.4161/cc.7.5.5427

[48] Blagoskolonny MV (2003) Cell senescence and hypermitogenic arrest. EMBO Rep 4: 358–362. 12671679 10.1038/sj.embor.embor806 PMC1319162

[49] Gauthier Y, Cario-Andre M, Lepreux S (2003) Melanocyte detachment after skin friction in non lesional skin of patients with generalized vitiligo. Br J Dermatol 148: 95–101. 12534601 10.1046/j.1365-2133.2003.05024.x

[50] Blagosklonny MV (2009) Aging-suppressants: cellular senescence (hyperactivation) and its pharmacologic deceleration. Cell Cycle 8: 1883–1887. 19448395 10.4161/cc.8.12.8815

[51] Blagosklonny MV (2011) Cell cycle arrest is not senescence. Aging 3: 94–101. 21297220 10.18632/aging.100281 PMC3082019

[52] Blagosklonny MV (2006) Cell senescence: hypertrophic arrest beyond the restriction point. J Cell Physiol 209: 592–597. 17001692 10.1002/jcp.20750

[53] Kostyuk VA, Potapovich AI, Cesareo E, Brescia S, Guerra L, et al (2010) Dysfunction of glutathione S- transferase leads to excess 4-hydroxy-2-nonenal and H(2)O(2) and impaired cytokine pattern in cultured keratinocytes and blood of vitiligo patients. Antioxid Redox Signal 13: 607–620. 20070240 10.1089/ars.2009.2976

[54] Pani A, Mandas A, Diaz G, Abete C, Cocco PL, et al (2009) Accumulation of neutral lipids in peripheral blood mononuclear cells as a distintive trait of Alzheimer patients and asymptomatic subjects at risk of disease. BMC Med 7: 66. 19883495 10.1186/1741-7015-7-66 PMC2777188

[55] Bondanza S, Maurelli R, Paterna P, Migliore E, Giacomo FD, et al (2007) Keratinocyte cultures from involved skin in vitiligo patients show an impaired in vitro behaviour. Pigment Cell Res 20: 288–300. 17630962 10.1111/j.1600-0749.2007.00385.x

[56] Slomonski A (2009) Neuroendocrine activity of the melanocyte. Exp Dermatol 18: 760–763. 19558501 10.1111/j.1600-0625.2009.00892.x PMC2773661

[57] Schallreuter KU, Tobin DJ, Panske A (2002) Decreased photodamage and low incidence of non-melanoma skin cancer in 136 sun exposed Caucasian patients with vitiligo. Dermatol 204: 194–201. 10.1159/000057881 12037447

[58] Feily A, Pazyar N (2011) Why vitiligo is associated with fewer risk of skin cancer?: providing a molecular mechanism. Arch Dermatol Res 303: 623–624. 21830160 10.1007/s00403-011-1165-5

[59] Michaloglou C, Vredeveld LC, Soengas MS, Denoyelle C, Kuilman T, et al (2005) BRAF600-associated senescence-like cell cycle arrest of human naevi. Nature 4: 720–724. 10.1038/nature03890 16079850

[60] Birol A (2006) Kisa U, Kurtipek GS, Kara F, Kocak M, et al (2006) Increased tumor necrosis factor alpha (TNF-alpha) and interleukin 1 alpha (IL1-alpha) levels in the lesional skin of patients with nonsegmental vitiligo. Int J Dermatol 45: 992–993. 16911396 10.1111/j.1365-4632.2006.02744.x

[61] Dumont P, Balbeur L, Remacle J, Dierick JF, Pascal T, et al (2000) Appearance of biomarkers of in vitro ageing after successive stimulation of WI-38 fibroblasts with IL-1alpha and TNF-alpha: senescence associated beta-galactosidase activity and morphotype transition. J Anat 197: 529–537. 11197525 10.1046/j.1469-7580.2000.19740529.x PMC1468167

[62] Gatza C, Moore L, Dumble M, Donehower LA (2007) Tumor suppressor dosage regulates stem cell dynamics during aging. Cell Cycle 6: 52–55. 17245110 10.4161/cc.6.1.3667

[63] Ruzankina Y, Asare A, Brown EJ (2008) Replicative stress, stem cells and aging. Mech Ageing Dev 129: 1313–1319. 10.1016/j.mad.2008.03.009 PMC2505188 18462780

