# Supplementary figures and images for: Vitiligo: A Possible Model of Degenerative Diseases
Source: PLoS One. 2013 Mar 26;8(3):e59782. doi: 10.1371/journal.pone.0059782 (PMC3608562; doi:10.1371/journal.pone.0059782)

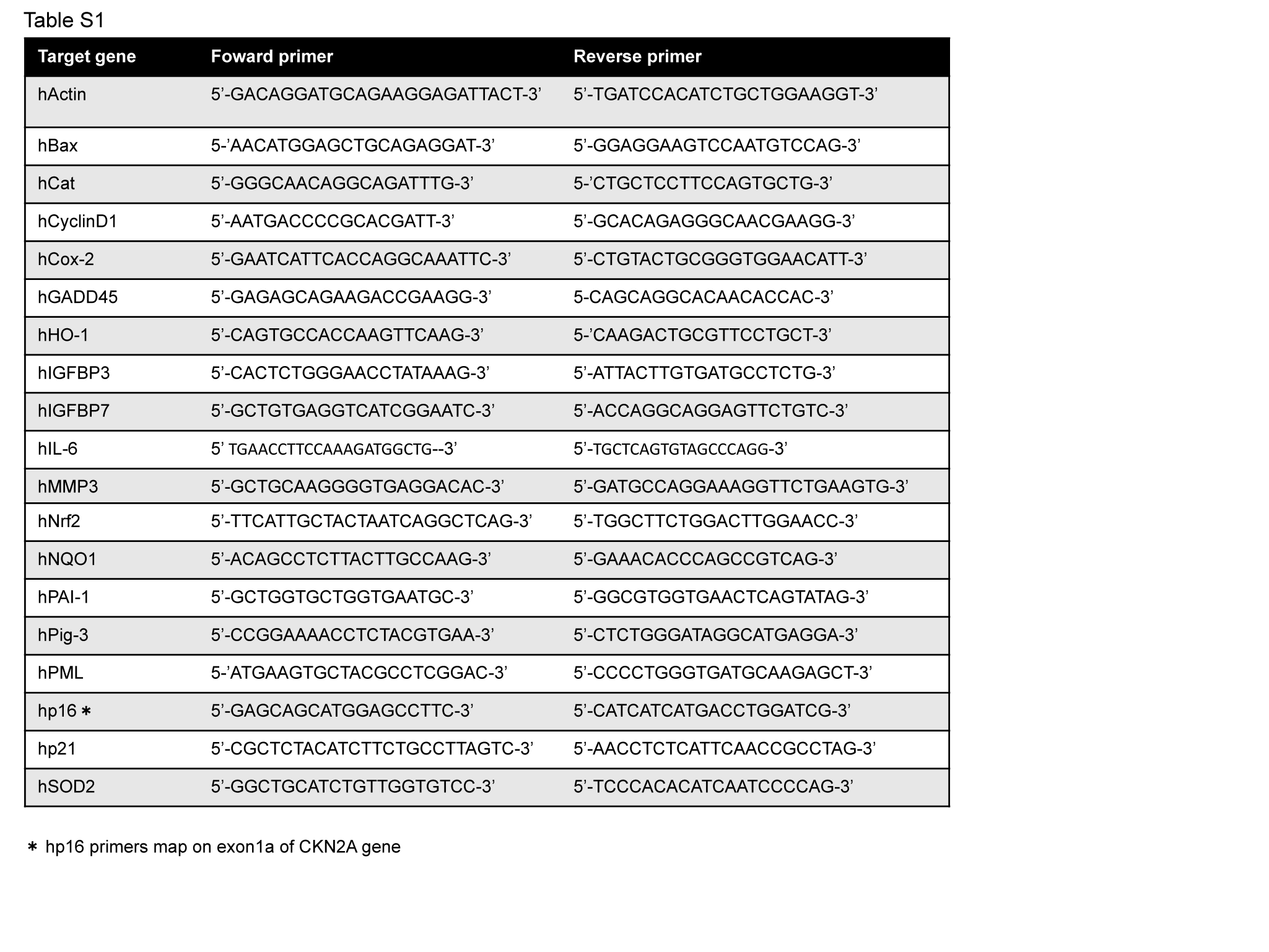

Supplement: Table S1 — List of primers used for quantitative real time PCR. Sequences of primers indicated with an F correspond to sense strands and with an R correspond to anti-sense. (TIF) [file pone.0059782.s001.tif]
